# Factors Influencing Postoperative Emergency Department Visits After Adenotonsillectomy in Pediatric Patients

**DOI:** 10.7759/cureus.104052

**Published:** 2026-02-22

**Authors:** Aseel A Alamoudi, Yasmeen Alhedyan, Ola Mohammed, Lenah A Shamsaddin, Lama S AlAlula, Fahad AlSaab

**Affiliations:** 1 College of Medicine, King Saud bin Abdulaziz University for Health Sciences, Riyadh, SAU; 2 Department of Otorhinolaryngology - Head and Neck Surgery, King Abdulaziz Medical City, Riyadh, SAU

**Keywords:** adenotonsillectomy, complications, emergency department visits, hospital stay, readmission

## Abstract

Purpose: This study investigates the relationship between preoperative factors and postoperative outcomes, particularly emergency department (ED) visits, in pediatric patients following adenotonsillectomy. It primarily focuses on the length of hospital stay in the procedure outcomes. The goal was to identify ways to reduce unexpected ED visits and poor outcomes after the procedure.

Method: A retrospective analysis was conducted on pediatric patients aged 2-14 who underwent adenotonsillectomy at King Abdullah Specialized Children’s Hospital (KASCH) between 2016 and 2023. Data were extracted from the BestCare database, including demographics, surgery-related factors, length of hospital stay, and postoperative outcomes such as ED visits, complications, and readmissions.

Results: The study included 700 adenotonsillectomy cases, with an average hospital stay of 1.0 days (1.0-9.0). Within 90 days post-surgery, there were 227 ED visits, with pain accounting for 29.5% of cases. Patients with a hospital stay of one day or more were more likely to experience ED visits (p = 0.030) and postoperative bleeding (p = 0.027), while those discharged in less than a day had lower readmission rates (p = 0.002). There was a direct correlation between the number of admission days followed by the procedure and the frequency of ED visits. Patients who had surgery for obstructive reasons or were prescribed sodium chloride nasal drops or penicillin at discharge (p = 0.014) had fewer postoperative ED visits (p = 0.010), respectively.

Conclusion: The study identified several preoperative factors and hospital stay durations that impact ED visit frequency after adenotonsillectomy. These findings underscore the importance of individualized postoperative care to reduce complications and ED visits in pediatric patients.

## Introduction

Adenotonsillectomy is one of the most commonly performed surgical procedures in pediatrics [1]. According to the American Academy of Otolaryngology - Head and Neck Surgery clinical practice guidelines, throat infections and breathing-related sleep disorders are the two most common indications for this procedure [2]. Although generally considered a safe procedure, adenotonsillectomy has been linked to several postoperative complications, leading to unplanned hospital readmissions and emergency department (ED) visits [3]. One consequence of unplanned readmissions is the worsening of pre-existing disorders and infections [4]. Furthermore, many readmissions after discharge are avoidable or unwarranted [5].

Studies show that a significant number of children present to the ED after adenotonsillectomy, with causes for ED visits varying slightly across studies. However, the most commonly reported reasons include hemorrhage, dehydration, nausea, and respiratory problems [6]. In a local study, the prevalence of unplanned readmissions at a tertiary pediatric hospital was 5.12%, with postoperative complications as the most common cause [7]. Hemorrhage is a leading cause of readmission following adenotonsillectomy [8]. Identifying the causes of ED visits and understanding the role of hospital stay length post-surgery can provide insight into the underlying causes of postoperative complications.

Recognizing preoperative risk factors for high ED visit rates is crucial for minimizing complications and optimizing patient care. Patient demographics may play a significant role in postoperative complications linked to increased ED visits. Age under three years has been identified as a factor that increases the risk of complications, leading to a higher likelihood of ED visits [9]. Research also shows a significantly higher risk of postoperative bleeding in children over six years, resulting in more frequent ED visits [6]. Similarly, medical conditions like obesity, severe sleep apnea, and neurological disorders might influence postoperative recovery and increase the risk of complications such as pulmonary edema or airway obstruction, which may lead to higher ED visit rates. Children with obstructive sleep apnea (OSA) are also at a higher risk of postoperative respiratory complications. Children with OSA are at increased risk for postoperative respiratory complications compared to the general pediatric population [10].

Additionally, previous ED visits for tonsil-related complaints have been linked to a higher likelihood of post-surgery ED revisits. Interestingly, the length of hospital stay after adenotonsillectomy has emerged as a potential risk factor for determining ED visit rate in pediatric patients [11]. Postoperative management following adenotonsillectomy commonly includes non-opioid analgesics such as acetaminophen and NSAIDs (nonsteroidal anti-inflammatory drugs) for pain control, with intraoperative dexamethasone routinely used to reduce postoperative pain and nausea [2]. Additional discharge medications may include saline nasal drops, antibiotics (such as penicillin or cephalosporins), or nasal decongestants, depending on institutional practice and clinical indication [12]. However, evidence suggests that variability in postoperative medication prescribing may influence recovery and healthcare utilization, including unplanned ED visits [3]. This highlights the importance of optimizing discharge planning and tailoring postoperative care to individual needs and risk factors. Despite existing research, identifying the full scope of preoperative risk factors and underlying causes associated with higher ED visits remains challenging. This study aimed to bridge that gap by investigating the independent and combined effects of various preoperative risk factors on the frequency of post-adenotonsillectomy ED visits in pediatric patients at a tertiary care hospital. The goal was to offer insights into preoperative risk stratification to improve postoperative care and potentially reduce unnecessary ED encounters. We hypothesized that the length of hospital stay following adenotonsillectomy is associated with the frequency and causes of ED visits.

This article was previously presented as an oral presentation at ER MNGHA Riyadh Day and awarded third place in February 2025 and as a poster presentation at ENT MNGHA Research Day in April 2025.

## Materials and methods

Study subjects and sample size

We retrospectively collected data from patient records at King Abdullah Specialized Children’s Hospital (KASCH) using the BestCare database. Ethical approval was obtained from the Institutional Review Board of King Abdullah International Medical Research Center (IRB No. 0237/24, Ref. No. RYD-24-419812-19116).To estimate the appropriate sample size, the expected sample size was calculated using RAOsoft software (Raosoft, Inc., Seattle, Washington; http://www.raosoft.com/samplesize.html). With a 95% confidence level and a 5% margin of error, the estimated sample size was 250 pediatric patients. The sample consisted of 700 consecutive pediatric patients aged 2-14 who underwent adenotonsillectomy, adenoidectomy, or tonsillectomy between 2016 and 2023. Patients who underwent multiple surgeries other than adenotonsillectomy simultaneously were excluded.

Data collection variables and tools

Demographic variables included age, sex, body mass index (BMI) (kg/m^2^), and comorbidities. Additionally, surgery-related variables were extracted, including indications for surgery, type of surgery, and surgeon's level. Information on the length of hospital stay was collected, encompassing the duration of the procedure, time in the post-anesthesia care unit, day care unit, and admission days following the procedure. Length of stay (LOS) was defined as the number of overnight stays in the hospital. Patients admitted and discharged on the same day were coded as LOS less than one day. Outcome variables assessed after adenotonsillectomy included ED visits, complications, postoperative care, discharge status, the need for further surgical interventions, and readmissions.

Statistical analysis

Data were analyzed using IBM SPSS Statistics for Windows, Version 26 (Released 2019; IBM Corp., Armonk, New York). Descriptive statistics were reported as numbers and percentages for categorical variables and medians (min-max) for continuous variables. The relationship between length of hospital stay and postoperative ED visits was assessed using the chi-square test and the Mann-Whitney U test. Furthermore, the relationship between postoperative ED visits and patients' demographic and clinical characteristics was analyzed using the same tests. Significant results were then subjected to multivariate regression analysis to identify important risk factors for postoperative ED visits, with corresponding odds ratios and confidence intervals reported. Statistical significance was set at p < 0.05. A large language model, ChatGPT (GPT-5.2) (2025), was used to create the graphs.

## Results

Patient characteristics

This study included 700 pediatric patients. Children aged 2-5 years constituted the majority (n = 332, 47.4%), with males being the dominant gender (n = 411, 58.7%). Most patients had a normal BMI (n = 563, 80.4%). The most common surgical indication was obstruction (n = 324, 46.3%). Adenotonsillectomy was the most frequently performed surgery (n = 531, 75.9%), with most surgeons being consultants (n = 451, 64.4%). Furthermore, the average lengths of surgery, stay in the post-anesthesia unit, and stay in the daycare unit were 28 minutes, 47 minutes, and 127 minutes, respectively. The average duration of postoperative admission was one day (Table 1).

**Table 1:** Demographic and clinical characteristics of the patients (n=700)

Study variables	N (%)
Age group	
· 2–5 years	332 (47.4%)
· 6–9 years	260 (37.1%)
· 10–14 years	108 (15.4%)
Gender	
· Male	411 (58.7%)
· Female	289 (41.3%)
Body mass index (BMI) level	
· Underweight: 18.5 kg/m² (<5th percentile)	33 (04.7%)
· Normal: 18.5–24.9 kg/m² (5th to <85th percentile)	563 (80.4%)
· Overweight: 25–29.9 kg/m² (85th to <95th percentile)	70 (10.0%)
· Obese: >30 kg/m² (≥95th percentile)	34 (04.9%)
Indication of surgery	
· Infective	153 (21.9%)
· Obstructive	324 (46.3%)
· Both (infective and obstructive)	223 (31.9%)
Type of surgery	
· Adenoidectomy	128 (18.3%)
· Adenotonsillectomy	531 (75.9%)
· Tonsillectomy	41 (05.9%)
Level of surgeon	
· Consultant	451 (64.4%)
· Consultant assistant	33 (04.7%)
· Consultant associate	39 (05.6%)
· Resident	148 (21.1%)
· Staff physician	17 (02.4%)
· Fellow	12 (01.7%)
	Median (min-max)
Length of surgery in minutes	28.0 (3.0–160.0)
Length of stay in post-anesthesia unit in minutes	47.0 (7.0–262.0)
Length of stay in daycare unit in minutes	127.0 (9.0–31,800)
Postoperative admission in days	1.0 (1.0–72.0)

Comorbidities and medications

**Figure 1:**
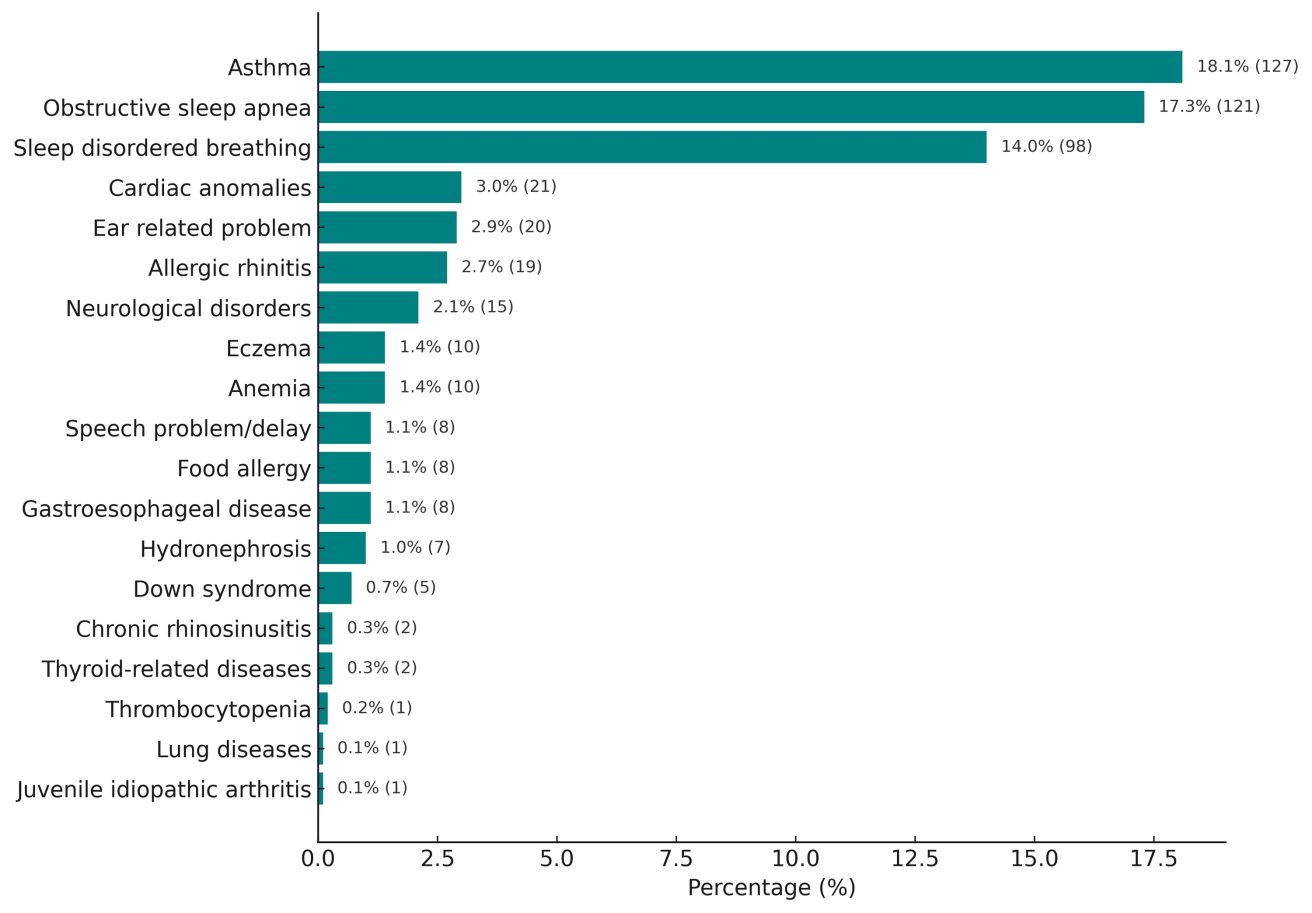
Patient comorbidities

As Figure 1 illustrates, the most frequently detected associated medical condition was asthma (18.1%, n = 127), followed by OSA (n = 121, 17.3%) and sleep-disordered breathing (n = 98, 14%).

**Figure 2:**
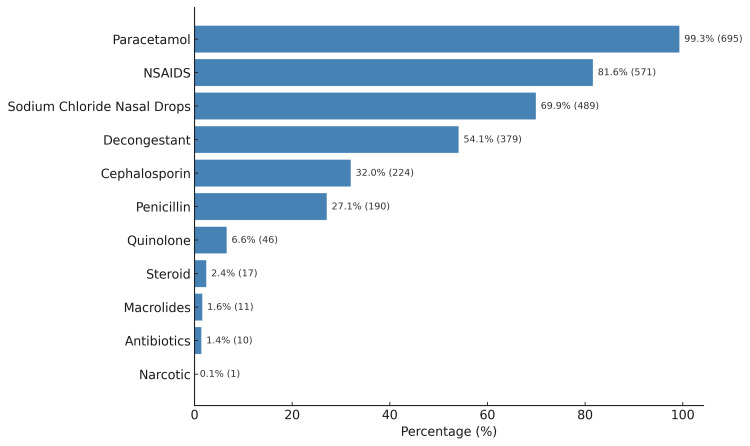
Discharge medications NSAIDs – Nonsteroidal Anti-Inflammatory Drugs

The three most commonly prescribed discharge medications were paracetamol (n = 695, 99.3%), NSAIDs (n = 571, 81.6%), and sodium chloride nasal drops (n = 489, 69.9%). Cephalosporins were the most frequently prescribed antibiotics (n = 224, 32%) (Figure 2).

Postoperative emergency room visits

There were 227 postoperative emergency room visits within 90 days of surgery. The leading cause of ED visits was pain (n = 67, 29.5%), followed by fever (n = 62, 27.3%) and poor oral intake (n = 57, 25.1%), with some patients presenting with more than one complaint. Admission was needed in 58 (25.6%) of the cases. The average frequency of ED visits was a single time (Table 2).

**Table 2:** Postoperative emergency room visit within 90 days (n = 227)

Variables	N (%)
Emergency room complaint	
· Pain	67 (29.5%)
· Fever	62 (27.3%)
· Poor oral intake	57 (25.1%)
· Nausea/vomiting	43 (18.9%)
· Upper respiratory complication	37 (16.3%)
· Reasons unrelated to adenotonsillectomy	32 (14.1%)
· Cough	30 (13.2%)
· Bleeding	30 (13.2%)
· Dehydration	10 (04.4%)
· Otitis media	06 (02.6%)
· Diarrhea	04 (01.8%)
· Lower respiratory complication	02 (0.90%)
· Rash	02 (0.90%)
· Cellulitis	01 (0.40%)
· Lymphadenopathy	01 (0.40%)
· Nasal discharge	01 (0.40%)
· Epistaxis	01 (0.40%)
Need for admission	
· No	169 (74.4%)
· Yes	58 (25.6%)
Need for surgical intervention	
· No	204 (89.9%)
· Yes	23 (10.1%)
Number of emergency room (ER) visits, median (min-max)	1.00 (1.00–15.00)
Duration of emergency visit in minutes, median (min-max)	24.0 (1.00–4320)

Association with demographics and clinical characteristics

The association between postoperative ED visits and the demographic and clinical characteristics of the patients is illustrated in Table 3. It was found that patients who sought postoperative ED visits were more likely to have undergone surgery due to infection (p < 0.001), have had adenotonsillectomy (p = 0.015), and be prescribed decongestants (p < 0.001) or cephalosporin (p < 0.001) upon discharge. In contrast, patients who received a prescription for sodium chloride nasal drops (p = 0.001) and penicillin (p = 0.001) at discharge had a lower risk of postoperative ED visits. As shown in Table 3, the LOS, including the procedure duration, post-anesthesia care unit, day care unit, and days of admission after the procedure, was not a statistically independent factor affecting the postoperative ED visit rate.

**Table 3:** Relationship between postoperative ER visits among the demographic and clinical characteristics of the patients (n = 700) ^§^ P-value has been calculated using the chi-square test. ^‡^ P-value has been calculated using the Mann-Whitney U test. ** Significant at p<0.05 level. Consultant consists of a consultant, an assistant consultant, and an associate consultant. Non-consultant consists of a resident, a staff physician, and a fellow.

Factor	Postoperative ER visit		P-value ^§^
	No N (%) (n = 393)	Yes N (%) (n=307)	
Age group			
· 2–5 years	178 (45.3%)	154 (50.2%)	0.352
· 6–9 years	149 (37.9%)	111 (36.2%)	
· 10–14 years	66 (16.8%)	42 (13.7%)	
Gender			
· Male	236 (60.1%)	175 (57.0%)	0.416
· Female	157 (39.9%)	132 (43.0%)	
BMI level			
· Normal or underweight	331 (84.2%)	265 (86.3%)	0.439
· Overweight or obese	62 (15.8%)	42 (13.7%)	
Indication of surgery			
· Infective	65 (16.5%)	88 (28.7%)	<0.001 **
· Obstructive	198 (50.4%)	126 (41.0%)	
· Both (infective and obstructive)	130 (33.1%)	93 (30.3%)	
Type of surgery			
· Adenoidectomy	85 (21.6%)	43 (14.0%)	0.015 **
· Adenotonsillectomy	282 (71.8%)	249 (81.1%)	
· Tonsillectomy	26 (06.6%)	15 (04.9%)	
Level of surgeon			
· Consultant	301 (76.6%)	222 (72.3%)	0.196
· Non-consultant	92 (23.4%)	85 (27.7%)	
Asthma			
· No	327 (83.2%)	246 (80.1%)	0.295
· Yes	66 (16.8%)	61 (19.9%)	
Obstructive sleep apnea			
· No	334 (85.0%)	245 (79.8%)	0.072
· Yes	59 (15.0%)	62 (20.2%)	
Sleep-disordered breathing			
· No	339 (86.3%)	263 (85.7%)	0.823
· Yes	54 (13.7%)	44 (14.3%)	
Discharge medications			
· Paracetamol	391 (99.5%)	304 (99.0%)	0.465
· NSAIDs	316 (80.4%)	255 (83.1%)	0.369
· Sodium chloride nasal drops	295 (75.1%)	194 (63.2%)	0.001 **
· Decongestants	190 (48.3%)	189 (61.6%)	<0.001 **
· Cephalosporin	104 (26.5%)	120 (39.1%)	<0.001 **
· Penicillin	126 (32.1%)	64 (20.8%)	0.001 **
	Mean ± SD	Mean ± SD	
Length of surgery in minutes	34.0 (6.0–53.0)	39.5 (18.0–78.0)	0.060
Length of stay in post-anesthesia unit in minutes	105 (35–220)	72.5 (30–235)	0.414
Length of stay in daycare unit in minutes	566 (104–2280)	727 (24–2160)	0.364
Postoperative admission in days	1.0 (1.0–9.0)	1.0 (1.0–72.0)	1.000

Multivariate regression analysis

Table 4 shows correlations between the indications for surgery and multiple factors related to the procedure, and postoperative ED visits were tested using multivariate regression analysis. The analysis revealed that patients who underwent surgery for an obstructive etiology (AOR = 0.539; 95% CI = 0.336-0.865; p = 0.010) and those who used sodium chloride nasal drops or penicillin at discharge (AOR = 0.600; 95% CI = 0.436-0.910; p = 0.014) were at a lower risk of having postoperative ER visits. In contrast, patients who used decongestants at discharge were at an increased risk of postoperative ED visits by at least 1.42 times (AOR = 1.419; 95% CI = 1.007-2.000; p = 0.045). No increase in postoperative ED visits was detected concerning the type of surgery or use of cephalosporins at discharge after adjusting for the regression model (p > 0.05).

**Table 4:** Multivariate regression analysis to determine the independent significant predictors of postoperative ER visits (n = 700) ** Significant at p<0.05 level. AOR – Adjusted Odds Ratio; CI – Confidence Interval; Ref – Reference.

Factor	AOR	95% CI	P-value
Indication of surgery			
· Infective	Ref	-	-
· ​​​​​​​Obstructive	0.539	0.336–0.865	0.010 **
· ​​​​​​​Both (infective and obstructive)	0.924	0.633–1.350	0.684
Type of surgery			
· ​​​​​​​Adenoidectomy	Ref	-	-
· ​​​​​​​Adenotonsillectomy	0.867	0.377–1.992	0.736
· ​​​​​​​Tonsillectomy	0.523	0.257–1.065	0.074
Use sodium chloride nasal drops at discharge			
· ​​​​​​​No	Ref	-	-
· ​​​​​​​Yes	0.630	0.436–0.910	0.014 **
Use of decongestants at discharge			
· ​​​​​​​No	Ref	-	-
· ​​​​​​​Yes	1.419	1.007–2.000	0.045 **
Use of cephalosporin at discharge			
· ​​​​​​​No	Ref	-	-
· ​​​​​​​Yes	1.366	0.896–2.082	0.147
Use of penicillin at discharge			
· ​​​​​​​No	Ref	-	-
· ​​​​​​​Yes	0.621	0.410–0.941	0.025 **

Length of hospital stay

Evidence strongly indicated that patients who had a hospital stay of one day or more were more likely to have postoperative ED visits (p = 0.030) and experience bleeding (p = 0.027) (Table 5). Interestingly, patients admitted for less than one day were less likely to be readmitted (p = 0.002). Additionally, no difference was detected in the need for additional surgical interventions compared to patients with a hospital stay of less than one day. Although the number of days of hospital stay showed a significant positive correlation with the rate of postoperative ED visits, there was no association between the frequency and duration of ED visits (Table 5).

**Table 5:** Length of hospital stay in relation to postoperative emergency room visit (n = 700) ^§^ P-value has been calculated using the chi-square test. ^‡^ P-value has been calculated using the Mann-Whitney U-test. ** Significant at p<0.05 level.

Variables	Length of Hospital Stay		P-value ^§^
	<1 day N (%) (n = 668)	≥1 day N (%) (n = 32)	
Postoperative emergency room visit			
· No	381 (57.0%)	12 (37.5%)	0.030 **
· Yes	287 (43.0%)	20 (62.5%)	
Emergency room complaint *			
· Fever	85 (29.6%)	04 (20.0%)	0.451
· Pain	78 (27.2%)	04 (20.0%)	0.607
· Reasons unrelated to adenotonsillectomy	65 (22.6%)	03 (15.0%)	0.582
· Nausea/vomiting	60 (20.9%)	03 (15.0%)	0.775
· Poor oral intake	57 (19.9%)	04 (20.0%)	1.000
· Upper respiratory complication	46 (16.0%)	02 (10.0%)	0.750
· Cough	32 (11.1%)	02 (10.0%)	1.000
· Bleeding	27 (09.4%)	05 (25.0%)	0.027 **
· Dehydration	13 (04.5%)	01 (05.0%)	1.000
· Diarrhea	11 (03.8%)	0	1.000
· Otitis media	07 (02.4%)	0	1.000
· Lower respiratory complication	02 (0.70%)	0	1.000
· Respiratory compromise	02 (0.70%)	0	1.000
· Cellulitis	02 (0.70%)	0	1.000
· Rash	02 (0.70%)	0	1.000
· Lymphadenopathy	01 (0.30%)	0	1.000
· Nasal discharge	01 (0.30%)	0	1.000
· Epistaxis	01 (0.30%)	0	1.000
Need for admission			
· No	230 (80.1%)	10 (50.0%)	0.002 **
· Yes	57 (19.9%)	10 (50.0%)	
Need for surgical intervention			
· No	264 (92.0%)	17 (85.0%)	0.234
· Yes	23 (08.0%)	03 (15.0%)	
	Median (min-max)	Median (min-max)	P-value ^‡^
Number of emergency room (ER) room visits	1.00 (1.00–15.00)	1.00 (1.00–6.00)	0.788
Duration of emergency visit in minutes	24.0 (1.00–10080)	19.5 (1.00–582)	0.890

## Discussion

This study investigated the rate of ED visits, their causes, and their association with the length of hospital stay following adenotonsillectomy in pediatric patients at a tertiary care hospital. The study population predominantly consisted of younger children, with nearly half of the patients aged 2-5 years, and a slight male predominance (58.7%). Pain, fever, and poor oral intake were the most commonly reported complaints during ED visits. Alsuhebani et al. [12] identified dehydration (22%), bleeding (19%), and pain (15%) as the most common reasons for associated return visits. Another study by Tran et al. [3] reported that hemorrhage (33%), unspecified surgical complications (21%), upper respiratory complications (14%), and infection (13%) were the most common diagnoses associated with unplanned ED visits.

The multivariate regression analysis, summarized in Table 4, revealed significant correlations between certain factors related to the surgical procedure and the likelihood of postoperative ED visits. Notably, patients who underwent adenotonsillectomy for obstructive causes had a significantly lower risk of returning to the ED (AOR = 0.539; 95% CI = 0.336 - 0.865; p = 0.010). This finding aligns with previous literature suggesting that patients undergoing adenotonsillectomy for infectious causes had higher odds of acute care revisits in the US [11]. This underscores the importance of tailoring postoperative care based on the underlying indications for surgery, as patients with infectious indications may require closer monitoring and more aggressive management to prevent complications that could lead to ED visits.

Regarding prescribed medications, those who used sodium chloride nasal drops or penicillin at discharge were at a lower risk of postoperative ED visits (AOR = 0.600; 95% CI = 0.436-0.910; p = 0.014). In contrast, Junaid et al. [12] reported that antibiotics did not result in a statistically significant decrease in postoperative complications necessitating ED visits. Moreover, intraoperative use of Otrivin prominently reduced postoperative respiratory compromise in pediatric patients undergoing adenotonsillectomy for OSA [14]. However, the present study found an increased risk of postoperative ED visits, at least 1.42 times (AOR = 1.419; 95% CI = 1.007-2.000; p = 0.045), in patients who received decongestants. No evident increase in postoperative ED visits was detected regarding the type of surgery and use of cephalosporins at discharge after adjusting for the regression model (p > 0.05).

Table 5 illustrates a significant relationship between the length of postoperative hospital stay and the frequency and reasons for ED visits after adenotonsillectomy. Specifically, patients with a hospital stay of one day or longer were notably more likely to visit the ED after the procedure, with an incidence rate of 62.5% (p = 0.030). The primary reason for ED visits was bleeding, which was strongly associated with an extended hospital stay (p = 0.027). These findings align with the research conducted by Tran et al., who observed that 4.8% of patients returning to the ED after adenotonsillectomy did so due to dehydration, while 5.0% were admitted for post-tonsillectomy hemorrhage [3]. There was a stark contrast regarding readmission rates based on the length of initial hospital stay. Patients discharged in less than one day had a readmission rate of 19.9% following an ED visit, whereas those with a hospital stay of one day or more had a significantly higher readmission rate of 50% (p=0.002). This pattern aligns with findings reported by Johnson RF, who noted that 80% of patients seen in the ED were treated without surgical intervention, resulting in considerably lower readmission rates of less than 2%. This suggests that while extended hospital stays are associated with a higher likelihood of ED visits and readmissions, many patients requiring ED care can be managed effectively without further hospitalization [15].

Limitations

The strengths of our study include detailed information on ED return rates and reasons for adenotonsillectomy visits. However, a few limitations should be noted. As this was a retrospective study, the surgical notes lacked clarity about the level of the operating surgeon. This led to potential underrepresentation of the distribution of surgeon experience statuses in our dataset. Notably, at our center, a considerable proportion of adenotonsillectomies are performed independently by residents without assistance from senior residents.

## Conclusions

This study provides valuable insights into the factors influencing postoperative ED visits among pediatric patients following adenotonsillectomy. Our findings suggest that the indications for surgery, postoperative medications, and length of hospital stay are significant predictors of ED revisits. The study revealed that an extended hospital stay correlated with higher rates of ED visits and readmissions, particularly due to postoperative bleeding. These findings emphasize the necessity for optimized discharge planning and close monitoring of patients with identified risk factors to minimize complications and reduce unnecessary healthcare utilization. Despite these insights, further research in diverse healthcare environments is needed to support and explore potential predictive factors for ED visits following adenotonsillectomy.
